# Evaluation of a Single Procedure Allowing the Isolation of Enteropathogenic *Yersinia* along with Other Bacterial Enteropathogens from Human Stools

**DOI:** 10.1371/journal.pone.0041176

**Published:** 2012-07-20

**Authors:** Cyril Savin, Alexandre Leclercq, Elisabeth Carniel

**Affiliations:** Yersinia Research Unit and National Reference Laboratory, Institut Pasteur, Paris, France; Charité, Campus Benjamin Franklin, Germany

## Abstract

Enteropathogenic *Yersinia* are among the most frequent agents of human diarrhea in temperate and cold countries. However, the incidence of yersiniosis is largely underestimated because of the peculiar growth characteristics of pathogenic *Yersinia*, which make their isolation from poly-contaminated samples difficult. The use of specific procedures for *Yersinia* isolation is required, but is expensive and time consuming, and therefore is not systematically performed in clinical pathology laboratories. A means to circumvent this problem would be to use a single procedure for the isolation of all bacterial enteropathogens. Since the Statens Serum Institut enteric medium (SSI) has been reported to allow the growth at 37°C of most Gram-negative bacteria, including *Yersinia*, our study aimed at evaluating its performances for *Yersinia* isolation, as compared to the commonly used *Yersinia*-specific semi-selective Cefsulodin-Irgasan-Novobiocin medium (CIN) incubated at 28°C. Our results show that *Yersinia pseudotuberculosis* growth was strongly inhibited on SSI at 37°C, and therefore that this medium is not suitable for the isolation of this species. All *Yersinia enterocolitica* strains tested grew on SSI, while some non-pathogenic *Yersinia* species were inhibited. The morphology of *Y. enterocolitica* colonies on SSI allowed their differentiation from various other Gram-negative bacteria commonly isolated from stool samples. However, in artificially contaminated human stools, the recovery of *Y. enterocolitica* colonies on SSI at 37°C was difficult and was 3 logs less sensitive than on CIN at 28°C. Therefore, despite its limitations, the use of a specific procedure (CIN incubated at 28°C) is still required for an efficient isolation of enteropathogenic *Yersinia* from stools.

## Introduction

The genus *Yersinia* belongs to the *Enterobacteriaceae* family and is composed of 17 species, of which two are human enteropathogens: *Yersinia pseudotuberculosis* and *Yersinia enterocolitica*. These two species have a worldwide distribution, with a higher incidence in temperate and cold countries [1], [2], [3]. Human yersinioses most often occur as sporadic cases, but clusters of cases or outbreaks have also been described [4], [5], [6], [7], [8]. Both species have an animal reservoir and a fecal-oral mode of transmission. Human infections commonly occur after ingestion of contaminated food or water, and less frequently through a direct contact with infected animals [9].

The usual clinical presentation associated with *Y. enterocolitica* and *Y. pseudotuberculosis* infections is an enterocolitis (diarrhea, abdominal pain, fever, and sometimes vomiting), which predominates in young children and is often self-limiting. More invasive and severe forms, such as septicemia, are not rare and occur predominantly in elderly patients with underlying conditions (hypersideremia, cirrhosis, diabetes) [10]. The most common secondary complications are reactive arthritis and erythema nodosum [6], [9], [11].

Although the incidence of *Y. pseudotuberculosis* is lower than that of *Y. enterocolitica* worldwide, the former species represents a major public health problem in some countries such as Japan or Russia [7], [12], [13], where it causes a particular and severe infection known as Izumi Fever or Far East Scarlet-Like Fever [14], [15]. *Y. enterocolitica* is a common etiological agent of enteritis in humans. This species is the third causative agent of diarrhea of bacterial origin in France [16] and Europe [17] after *Campylobacter* and *Salmonella*. The incidence of human yersiniosis has been estimated to be 16 per 100,000 inhabitants in France [18], 1.65 per 100,000 in Europe [17], and 0.3 per 100,000 in the USA [19], but is probably largely underestimated.

A survey conducted among a sample of 483 clinical pathology laboratories covering the French territory [18] evidenced several reasons for the under evaluation of the *Yersinia* risk. One of them was the absence of stool culture prescriptions for patients presenting with mild enteritis. A major factor accounting for this underestimation was the difficulty to recover *Yersinia* strains from poly-contaminated biological samples such as stools. Indeed, *Yersinia* strains differ from other enterobacteria by a slower growth rate and an optimal growth temperature of 28°C instead of 37°C. Therefore, stools cultures performed under conditions suitable for most enteropathogens (incubation at 37°C for 24 h) are not effective for the recovery of *Yersinia* colonies. Finally, because declaration of human yersinioses is not mandatory in France, the number of cases reported to the Reference Laboratory does not reflect accurately the number of diagnosed human cases.

To improve the isolation rate of *Yersinia* strains, various enrichment procedures have been described. One of the first was a cold enrichment (4°C) in Phosphate Buffered Saline (PBS), but this procedure was long (1 to 3 weeks) and favored the growth of non-pathogenic species [20], [21]. Enrichment in Irgasan-Ticarcilline-Chlorate (ITC) broth improved the recovery of *Y. enterocolitica* biotype 4, but was not suitable for biotype 2 strains [22]. The use of Bile-Oxalate-Sorbose (BOS) increased the isolation rate of *Y. enterocolitica* biotype 1B, but these strains are extremely rare in most countries outside the USA [9], [23]. The European norm ISO 10273:2003 is currently considered to give the best recovery rate for enteropathogenic *Yersinia* from the food chain and primary production samples such as animal feces. It consists in two methods: (i) enrichment in ITC broth for 48 h at 25°C followed by isolation on Salmonella-Shigella medium with sodium deoxycholate and calcium chloride (SSDC) selective medium, and (ii) enrichment in Peptone-Sorbitol-Bile salt (PSB) broth for 24 to 48 h at 22–25°C [24] followed or not by an alkali treatment (0.5 ml of PSB broth transferred to 4.5 ml of 0.5% KOH solution and mixed for 20 s) before isolation on Cefsulodin-Irgasan-Novobiocin (CIN) selective medium. However, this procedure is time and labor intensive consuming and therefore it is not routinely done in most clinical pathology laboratories.

Several attempts have been made to develop *Yersinia* selective media. The addition of sodium deoxycholate and CaCl_2_ to Salmonella-Shigella agar (SSDC agar) proved to be useful for *Y. enterocolitica* recovery, but when combined with an enrichment procedure [22]. Other media such as BABY4 [25], VYE agar [26], KV202 agar [27], YeCM [28] and recently YeC4 [29] also improved the isolation of *Y. enterocolitica*, but growth of *Y. pseudotuberculosis* strains was inhibited or not tested. Moreover, none of these media are marketed, which is problematic or appalling for a routine use by clinical pathology laboratories [23].

A semi-selective medium known as CIN [30] has been developed and manufactured for the specific isolation of *Yersinia* species. Seeded CIN agar plates are examined after 24 h and 48 h incubation at 28°C for the presence of characteristic *Yersinia* colonies (deep red center with a sharp border surrounded by a translucent zone). As this medium is strongly inhibitory for many bacterial species, it significantly improved the rate of *Yersinia* isolation. However, CIN impairs the growth of some enteropathogenic strains such as *Y. pseudotuberculosis* [31]. To date, CIN agar is the most widely used medium for *Yersinia* isolation in clinical pathology laboratories, but the French survey [18] showed that it is far from being systematically used because of the additional cost and the increase in workload resulting from the use of a specific medium at a specific temperature for the isolation of enteropathogenic *Yersinia* strains.

A medium suited for routine stool culture practices would ideally allow the growth and isolation of all pathogenic bacteria (including *Yersinia*), using a standard and unique procedure, while being commercially available. The recently developed and manufactured Statens Serum Institut Enteric Medium (SSI) was shown to allow the growth of all Gram-negative bacteria (except *Campylobacter*), including the 12 *Yersinia* spp. tested from fecal samples incubated for 24 h at 37°C [32]. This medium could detect six biochemical reactions (H_2_S production, lactose fermentation, phenylalanine deaminase reaction, indole production, metallic sheen reaction for producing H_2_S colonies, and rough transformation) [23], [32], and was reported to make possible the distinction of the 12 *Yersinia* spp. tested (translucent colonies resembling pearls on a string) from other enterobacteria.

The aim of this study was to determine whether the SSI enteric medium (designated SSI here) could, under standard conditions used by clinical pathology laboratories for stool cultures, allow an efficient detection of all enteropathogenic *Yersinia*.

## Materials and Methods

### Ethics Statement

No ethics approval was necessary because the human sample was recovered from the first author of the manuscript.

A Verbal informed consent of the first author was obtained. No written consent was obtained because the first author recovered its human sample and he performed the experiments on it.

### Bacterial strains

The 94 *Yersinia* strains studied are listed in Table S1. They were taken from the collection of the *Yersinia* Research Unit and National Reference Laboratory (Institut Pasteur, Paris, France). The non-*Yersinia* enterobacteria listed in Table 1 were kindly provided by Prof. P. A. D. Grimont (Institut Pasteur, Paris, France). All strains were grown overnight in 10 ml LB broth under agitation at 28°C (*Yersinia*) or 37°C (non-*Yersinia* species) before performing the assays.

**Table 1:** Colony morphology of various *Yersinia* and non-*Yersinia* enterobacteria grown for 24 h on SSI.

			28°C			37°C		
Species	Strain number	Bioserotype	Diameter (mm)	Edge	Center	Diameter (mm)	Edge	Center
Pathogenic *Y. enterocolitica*								
	IP17451	1B/O:8	1	Translucent	Pink	1–1.5	Translucent	Pink
	IP29388	2/O:5,27	Pinpoint	Translucent	Translucent	0.5	Pale pink	Pale pink
	IP29497	2/O:9	0.5	Pale pink	Pink	0.5–1.5	Pale pink	Dark pink
	IP29159	3/O:3	0.5–1	Pale pink	Pink	1–1.5	Pale pink	Pink
	IP29464	4/O:3	0.5	Translucent	Pale pink	0.5	Pale pink	Pink
	IP29492	4/O:3	0.5–1	Pale pink	Pink	0.5–1	Pale pink	Dark pink
Non-pathogenic *Yersinia*								
*Y. enterocolitica*	IP29468	1A/O:18	1.5–2	Pink	Pink	2	Pale pink	Pink
*Y. frederiksenii*	IP29447	NAG	1–1.5	Pale pink	Pink	1.5–2	Pale Pink	Pink
*Y. kristensenii*	IP29443	O:16-16,29	1	Pale pink	Pink	1–1.5	Pale Pink	Dark pink
Other enterobacteria								
*Proteus vulgaris*	R-R-1	NA	2	Translucent	Grey, large	2.5–3	Translucent	Grey, large
*Shigella dysenteriae*	NCDC2783 71	NA	1.5–2	Pale white	Pale white	3	Pale white	Pale white
*Cronobacter sakazakii*	4562 70	NA	0.5–1	Translucent	Pale pink	0.5–1	Pale pink	Pink
*Klebsiella oxytoca*	282	NA	2–2.5	Pink	Pink	3	Pink	Pink
*Citrobacter freundii*	460.61	NA	0.5–1.5	Translucent	Black	1–1.5	Translucent	Black
*Salmonella* Typhimurium	LT2	NA	1–1.5	Translucent	Black	3–4	Translucent	Black, large
*Escherichia coli*	K12	NA	1.5–2	Dark pink	Dark pink	3	Dark pink	Dark pink

NAG: non-agglutinable; NA: not applicable.

### Culture media

Luria-Bertani (LB) broth was used to grow bacteria prior to plating. Plating media used in this study were the SSI enteric medium (SSI, Statens Serum Institut, Denmark), the Cefsulodin-Irgasan-Noviobiocin agar (CIN, Merck, Switzerland), and the non-selective Trypticase Soy Agar (TSA, Oxoid, France) as a control medium.

### Quantification of bacterial growth

Overnight cultures of *Y. enterocolitica* and *pseudotuberculosis* strains were adjusted to an optical density at 600 nm of 1 (corresponding approximately to 5×10^8^ colony forming unit (cfu)/ml) and serially diluted to an estimated concentration of 10^3^ cfu/ml. One hundred µl of this bacterial suspension were plated in quadruplicate onto TSA, CIN and SSI plates. For each of the three media, two plates were incubated at 28°C and the other two at 37°C. The number of colonies was counted after 24 h and 48 h and the average number of cfu on duplicated plates incubated at the same temperature was calculated. Results were expressed as percentages of mean cfu on CIN/TSA and SSI/TSA at each temperature.

### Semiquantitative growth of 92 *Yersinia* strains

After overnight growth in LB broth, 200 µl of bacterial cultures were deposited in the 12 wells of the first horizontal row of a 96-wells microplate. Ten-fold serial dilutions in LB broth were vertically performed as schematized on figure S1. TSA, CIN and SSI plates were seeded with 5 µl of the bacterial suspension from each microwell and incubated for 48 h at 28°C and 37°C (Fig. S1). The dilution limit was defined as the highest 10-fold dilution of the bacterial suspension that still yielded colonies on agar plates. The results were expressed as the difference in log number between the dilution limits on TSA and on either CIN or SSI.

### Isolation assessment

Overnight cultures of *Yersinia* strains and various enterobacteria were adjusted to 10^3^ cfu/ml, and 100 µl of the bacterial suspensions were plated on SSI plates. To compare the colony morphology of *Y. enterocolitica* with those of some other enterobacteria, mixtures containing 50 µl of *Y. enterocolitica* IP29492 and 50 µl of another enterobacteria were plated in duplicate onto CIN and SSI plates. One plate each of the two media was incubated at 28°C and the other one at 37°C. The plates were examined after 24 h and 48 h of incubation.

### Detection of *Y. enterocolitica* in artificially contaminated stool cultures

The equivalent of a pea (corresponding approximately to 1 g) of feces from a healthy individual was diluted in 10 ml of saline. An overnight culture in LB broth at 28°C of *Y. enterocolitica* IP29492 of bioserotype 4/O:3 was adjusted to ≈10^8^ cfu/ml and 10-fold serially diluted in saline. Twenty µl of bacterial suspensions containing from 10^8^ to 10^4^ cfu/ml were added to 1 ml of saline. Ten µl of each suspension were plated in duplicate on TSA plates and incubated at 28°C or 37°C to determine the exact number of *Y. enterocolitica* cfu. In parallel, twenty µl of the same bacterial suspensions were added to 1 ml of feces suspensions. Ten µl of each stool suspension were streaked in duplicate on SSI and CIN plates that were incubated at 28°C or 37°C. Bacterial colonies were examined after 24 h and 48 h of growth. *Yersinia*-resembling colonies were picked and agglutinated with an anti-O:3 specific antiserum (National Reference Laboratory, Institut Pasteur) to confirm their identification.

## Results

### Growth of enteropathogenic *Yersinia* on CIN and SSI, as compared to TSA

First we wanted to evaluate the capacity of SSI to allow the growth of enteropathogenic *Yersinia*, as compared to that of CIN. For this purpose, bacterial suspensions corresponding to approximately 100 cfu of eight strains of *Y. pseudotuberculosis* or *Y. enterocolitica* of various bioserotypes were plated in parallel and in quadruplicate on TSA, CIN and SSI plates and incubated at 28°C or 37°C. Percentages of the mean cfu on CIN/TSA or SSI/TSA that were comprised between ≈80 and 120% were considered to indicate growth efficiency on CIN or SSI similar to that on TSA (100%).

At 28°C, most *Y. enterocolitica* and the two *Y. pseudotuberculosis* strains tested grew on CIN and SSI as well as on TSA (Table 2). Exceptions were the two 3/O:3 strains, which both showed a growth defect on CIN, and one of these two strains (IP28877) had a severe growth deficiency on SSI. At 37°C, the growth defect observed for the two 3/O:3 strains disappeared on CIN, but remained on SSI for strain IP28877 (Table 2). Furthermore, this incubation temperature had some inhibitory effect on the 2/O:9 strain grown on CIN but not on SSI. In contrast, the growth at 37°C of the two *Y. pseudotuberculosis* strains was severely impaired on SSI but not on CIN (Table 2). Therefore, both CIN and SSI exerted an inhibitory activity on a few strains, and this effect was temperature-dependent.

**Table 2:** Growth at different temperatures of enteropathogenic *Yersinia* strains on CIN and SSI, as compared to TSA.

				% of cfu						
				28°C		37°C		37°C/28°C		
Species	Strain #	Biotype	Serotype	CIN/TSA	SSI/TSA	CIN/TSA	SSI/TSA	TSA	CIN	SSI
*Y. enterocolitica*	Ye8081	1B	O:8	83.2	100.0	82.6	99.8	104.2	103.3	104.0
	IP24083	2	O:9	97.8	96.2	44.8	99.5	100.5	46.1	104.0
	IP29159	3	O:3	43.7	81.6	118.0	139.8	81.0	218.8	138.8
	IP28877	3	O:3	68.7	6.8	98.4	9.4	88.1	126.2	121.1
	IP29534	4	O:3	92.8	103.0	79.6	126.7	95.3	81.7	117.3
	IP29464	4	O:3	137.1	122.1	122.7	118.7	110.0	98.5	107.0
*Y. pseudotuberculosis*	IP33426	NA	I	108.8	107.4	96.1	0.0	107.9	95.3	0.0
	IP32544	NA	III	112.0	103.3	116.4	0.5	81.9	85.2	0.4

NA: not applicable.

To differentiate the impact of the medium from that of the incubation temperature, the results were expressed as the percentage of growth on each medium at 37°C versus 28°C. No impact of the incubation temperature was noted for bacteria grown on TSA plates (percentages comprised between 80 and 120%, Table 2). On CIN, incubation at 37°C was less favorable than at 28°C for the 2/O:9 strain, while the opposite effect was observed for one 3/O:3 strain (IP29159). On SSI, this 3/O:3 strain also grew better at 37°C than at 28°C. The impact of the temperature was the most striking on *Y. pseudotuberculosis*, as an almost complete inhibition was observed for the two strains grown at 37°C on SSI. In contrast, the incubation temperature did not alter *Y. pseudotuberculosis* growth on CIN (Table 2).

Altogether our results indicate that both CIN and SSI allow the growth of various enteropathogenic *Yersinia* strains, but that some strain-dependent and temperature-dependent inhibitory activities are observed with both media.

### Semiquantitative growth of 92 *Yersinia* strains

To further evaluate the efficiency of SSI to allow *Yersinia* growth as compared to CIN, a panel of 92 strains (Table S1) belonging to the two enteropathogenic species *Y. enterocolitica* (49 strains of biotypes 1B - 5) and *Y. pseudotuberculosis* (24 strains of the most common serotypes I–III), as well as non-pathogenic *Yersinia* isolates (19 strains) belonging to eight different species were selected. To allow the screening of a large number of isolates, we designed a simplified procedure described in Material and Methods and schematized on figure S1. The highest 10-fold dilution of the bacterial suspension that still allowed to observe colonies (dilution limit) was recorded. Results were expressed as the difference in log number between the dilution limits on TSA and on either CIN or SSI. Therefore, a ‘0’ indicated the absence of growth inhibitory effect while a ‘2’ indicated a 100 times less efficient growth on the tested medium than on TSA. This method is less sensitive than the previous one, but allows the simple and rapid screening of a much larger number of isolates. To determine the influence of the incubation temperature on growth efficiency, the experiments were done in parallel on two plates that were incubated at 28°C or 37°C.

All pathogenic *Y. enterocolitica* grew efficiently on CIN and SSI, at both 28°C and 37°C (Table 3). The only exception was one 3/O:3 strain (IP28877) whose growth was impaired on SSI at 28°C and on CIN at 37°C. Non-pathogenic *Yersinia* strains were able to grow normally on CIN at both 28°C and 37°C. In contrast, SSI had a moderate inhibitory effect on the growth of one *Y. mollaretii* and one *Y. aldovae* isolate at 28°C, and had a moderate to severe inhibitory activity at 37°C on the *Y. bercovieri, Y. mollaretii* and *Y. intermedia* isolates tested (Table 3).

**Table 3:** Growth of 92 *Yersinia* strains on SSI and CIN as compared to TSA (control medium).

				Inhibitory effect of CIN or SSI^a^			
				28°C		37°C	
Species	Biotype	Serotype	Number of strains	CIN	SSI	CIN	SSI
Pathogenic *Yersinia*							
*Y. enterocolitica*	4	O:3	13	0	0	0	0
	2	O:9	13	0	0	0	0
	2	O:5,27	6	0	0	0	0
	3	O:3	6	0	0–3	0–1	0
	5	O:1,2,3	1	0	0	0	0
	1B	Various	10	0	0	0	0
*Y. pseudotuberculosis*	NA	I	13	0–1	0–1	1	4–5
	NA	II	6	0–4	0	0–4	3–4
	NA	III	5	0–2	0–4	0–4	2–4
Non-pathogenic *Yersinia*							
*Y. enterocolitica*	1A	Various	6	0	0	0	0
*Y. frederiksenii*	NA	Various	2	0	0	0	0
*Y. kristensenii*	NA	Various	2	0	0	0	0
*Y. rohdei*	NA	NAG	1	0	0	0	0
*Y. bercovieri*	NA	Various	2	0	0	0	4–5
*Y. mollaretii*	NA	Various	2	0	0–1	0	1–5
*Y. intermedia*	2	Various	3	0	0	0	3–6
*Y. aldovae*	NA	NAG	1	0	1	0	0

a : the results are expressed as the difference of the log of the dilution limits between TSA and either CIN or SSI.

NA: Not applicable; NAG: non-agglutinable.

Of note, both media impaired to different extents the growth of *Y. pseudotuberculosis* (Table 3). This effect was markedly strain and temperature-dependent, as some isolates grew as well on these media as on TSA plates, while others exhibited up to 100,000 fold growth inhibition. A further analysis of this effect showed that it was partly serotype-dependent (Table S2). At 28°C, all strains of serotype I grew similarly on CIN and SSI, exhibiting a mild growth inhibition (1 log for all but one), in comparison with TSA. SSI was less inhibitory than CIN at 28°C for serotype II and III isolates, as out of the 11 strains tested, nine grew normally on SSI, and seven on CIN (Table S2). The most striking effect was observed at 37°C. On CIN, strains of serotype I grew as well as at 28°C, exhibiting a moderate inhibition (1 log for all, Table S2), but serotype II and III isolates were more affected, with 7/11 strains displaying a growth defect, which was severe (3 to 4 log decrease) for most of them (Table S2). The effect of the temperature was even harsher on SSI. Incubation at 37°C strongly affected the growth of all *Y. pseudotuberculosis* isolates, whatever their serotype, leading to a 100 to 100,000 fold growth inhibition (Table S2).

Therefore, SSI incubated at 37°C exerted more inhibitory effects than CIN on some non-pathogenic *Yersinia* species. SSI had also the advantage of being less restrictive than CIN for the growth of pathogenic *Y. enterocolitica* strains at 37°C. However, SSI impaired dramatically, and much more severely than CIN, the growth at 37°C of all *Y. pseudotuberculosis* strains tested.

### Comparison of the morphology of *Yersinia* and some other common enterobacteria colonies on SSI

The shape, size and color of pathogenic *Y. enterocolitica* colonies grown on SSI plates at 28°C or 37°C, were then compared to those of non-pathogenic *Yersinia* and other common enterobacteria isolated from human stools. The shape of the colonies of all *Yersinia* and non-*Yersinia* strains tested was roughly similar (round) at both temperatures, so this characteristic could not be used as a distinctive trait.

After 24 h of growth, the size of the colonies of pathogenic *Y. enterocolitica* was small (pinpoint to 1 mm in diameter) at 28°C, and tended to be slightly larger at 37°C (0.5 to 1.5 mm, Table 1), with a certain heterogeneity in colony size. Other non-pathogenic *Yersinia* and enterobacteria tested had most often larger colonies at the two temperatures, with the exception of *Y. kristensenii*, *Cronobacter sakazakii* and *Citrobacter freundii*, whose colonies had diameters comparable to those of pathogenic *Y. enterocolitica* at both temperatures (Table 1).

**Figure 1:**
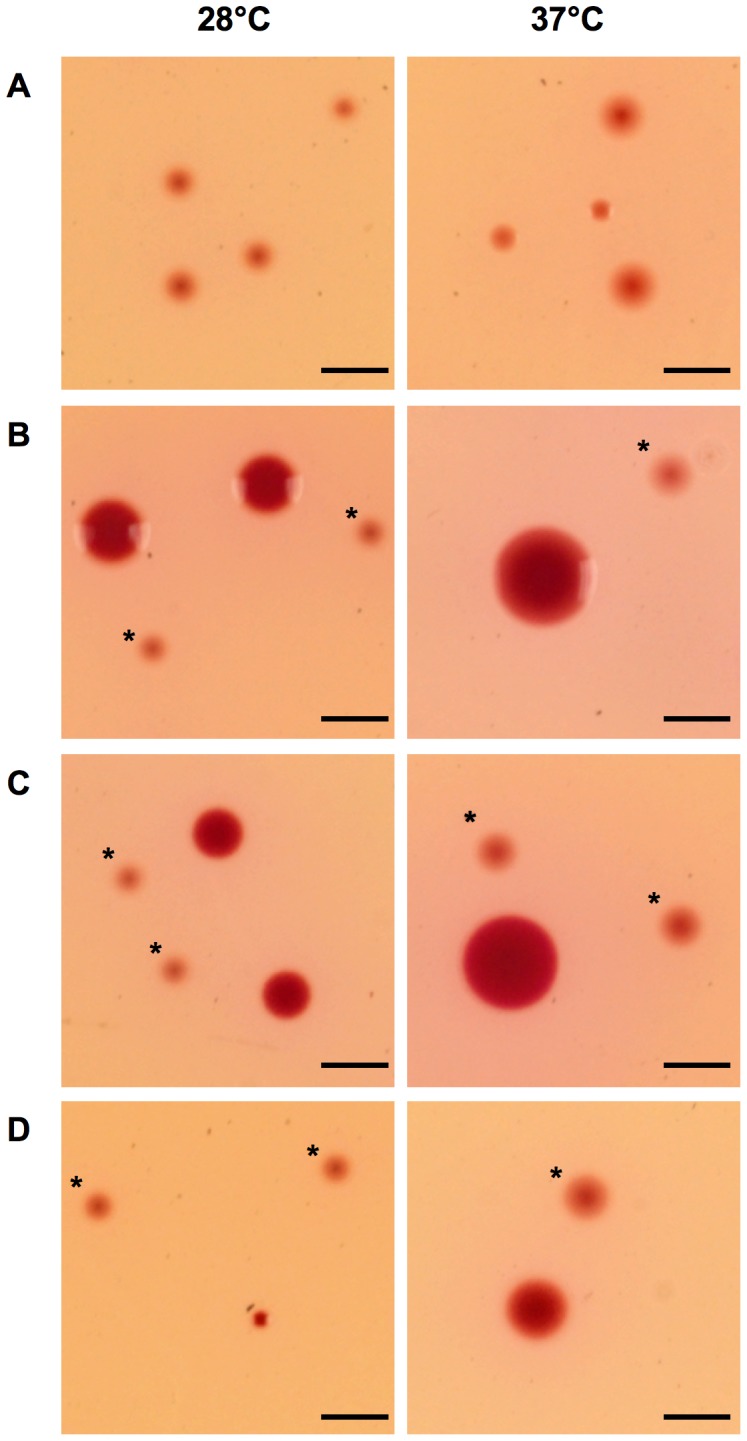
Colonies of *Y. enterocolitica* alone or with enterobacteria after 24 h of growth on SSI at 28°C and 37°C. *Y. enterocolitica* 4/O:3 (strain IP29492) alone (A), or mixed with *K. oxytoca* (B), *E. coli*. (C), *C. sakazakii* (D). Colonies of *Y. enterocolitica* are indicated with a star on mixed plates. The black bar represents 2 mm.

*Yersinia* colonies (enteropathogenic and non-pathogenic strains) were characterized by a translucent or pale pink edge with a more or less intense pink center at both temperatures (Fig. 1A and Table 1). Some other enterobacteria could be easily distinguished from *Yersinia* as their colonies exhibited a grey (*Proteus vulgaris*), pale white (*Shigella dysenteriae*), or black (*C. freundii* and *Salmonella* Typhimurium) center. However, *C. sakazakii*, *Klebsiella oxytoca* and *Escherichia coli* displayed pink colonies, which could resemble those of *Y. enterocolitica*. To determine whether colonies of these three enterobacteria could be distinguished from those of pathogenic *Y. enterocolitica*, one strain of bioserotype 4/O:3 (IP29492) was mixed with either *E. coli, C. sakazakii* or *K. oxytoca* and streaked on SSI. As shown on figure 1B & 1C, the darker pink color and the larger size of the colonies of *E. coli* and *K. oxytoca* allowed their differentiation from those of *Y. enterocolitica* at both 28°C and 37°C. In contrast, *C. sakazakii* could hardly be distinguished from *Y. enterocolitica* at 28°C (Fig. 1D): colonies were of similar size and although the center of the *C. sakazakii* colonies had a more intense pink color, this difference was minor. However, at 37°C, the colonies were more easily distinguished because the size of the *C. sakazakii* colonies was larger than those of *Y. enterocolitica* (Fig. 1D).

An incubation of the plates for an additional 24 h (48 h in total) slightly enhanced the intensity of the colors and the sizes of the colonies of all strains in the same proportion (data not shown) and therefore did not significantly improve the distinction of *Y. enterocolitica* from other enterobacteria.

Therefore *Y. enterocolitica* colonies could be distinguished from those of the other enterobacteria tested after 24 h of growth at 37°C on SSI.

### Comparative efficiency of CIN and SSI to recover pathogenic *Y. enterocolitica* in a fecal flora

Since SSI does not have the inhibitory effect of CIN on various bacterial species, a major question was whether the presence of numerous bacteria composing the gut microflora could still allow the detection of *Y. enterocolitica* on SSI plates. For this purpose, a normal human stool was suspended in saline and 10 µl of this suspension were streaked on SSI or CIN. Approximately 5 colonies/plate having a morphology clearly different from that of *Y. enterocolitica* were counted on CIN. In contrast, a dense bacterial growth was observed on SSI, confirming the lower selective effect of SSI.

The stool suspension was then artificially contaminated with 10-fold serial dilutions of *Y. enterocolitica* IP29492 and 10 µl of various dilutions containing from 3×10^4^ to 3 cfu/10 µl (as determined after enumeration on TSA) were streaked on CIN or SSI. On CIN, almost all colonies had a typical *Yersinia* morphology (Fig. S2), and their numbers corresponded to those of the inocula. Their identification as *Y. enterocolitica* 4/O:3 was confirmed by the fact that all picked colonies agglutinated with the anti-O:3 antiserum. On SSI, the stool suspensions contaminated with the highest *Y. enterocolitica* concentrations (3×10^4^ and 3×10^3^ cfu per plate) allowed the isolation of 18 *Yersinia*-like colonies, and half of them were confirmed to be *Y. enterocolitica* after anti-O:3 agglutination (Fig. S2). At lower bacterial concentrations (3×10^2^ to 3 cfu per plate), most of the isolated colonies had a morphology not characteristic of *Y. enterocolitica*, and those that were picked did not agglutinate with the anti-O:3 antiserum. Similar results were obtained after incubation of the plates at 28°C or 37°C, and after examination after 24 h or 48 h.

Altogether our results indicate that the recovery on SSI of *Y. enterocolitica* from stool samples requires bacterial concentrations ≥3×10^6^ *Yersinia* cfu/g of feces and is hardly possible at lower bacterial numbers.

## Discussion

Yersiniosis is a common cause of gastrointestinal symptoms in many countries worldwide, but its incidence is largely underestimated. A recent survey conducted among 483 French medical laboratories identified several reasons for this underestimation, but one of the major factors was the difficulty to recover *Yersinia* strains from poly-contaminated biological samples such as stools [18]. Because of their peculiar growth characteristics (slow growth rate and optimal temperature of 28°C), stool cultures performed under conditions suitable for most enteropathogens (incubation at 37°C for 24 h) hardly allow the recovery of *Yersinia* colonies on non-selective plates.

Various enrichment procedures have been proposed to improve the recovery rate of *Yersinia* from poly-contaminated samples [9], [20], [21], [22], [23], [24], [33], but they delay the etiological diagnosis, they require the use of specific broths that are not always commercially available, they are inhibitory for some enteropathogenic strains, and they are time and labor intensive. For all these reasons, they are not routinely carried out in most medical laboratories. Currently, the most commonly used procedure is to directly streak stool suspensions on CIN plates [30] that are incubated at 28°C and examined after 24 h and 48 h. Although the search for *Yersinia* from human diarrheal stools is compulsory in France (French Official Journal – November 2003), the recent survey revealed that 45% of the clinical pathology laboratories do not systematically perform specific procedures for the isolation of *Yersinia* [18]. From a practical point of view, the routine use of CIN by clinical pathology laboratories is hampered by the necessity to purchase a *Yersinia*-specific medium (additional cost), and to process a set of plates at a particular temperature (28°C) for the detection of enteropathogenic *Yersinia* (increased work load).

A means to circumvent this practical problem would be to use a unique and simple procedure suitable for the detection of enteropathogenic bacteria. This would mean utilizing a semi-selective medium that would be commercially available and would allow the selective growth of all enteropathogenic Gram-negative bacteria (including *Yersinia*). The SSI enteric medium has been reported to have these characteristics [32]. This marketed medium was shown to allow an efficient growth of all Gram-negative bacteria, except *Campylobacter*, and the differentiation of enterobacteria commonly isolated from stool sample, based on the color and size of the isolated colonies. The aim of this study was thus to determine whether it could be possible to replace the *Yersinia*-specific isolation procedure based on CIN by a procedure applicable to all enteropathogenic bacteria (i.e. streaking on SSI and incubation at 37°C for 24 h).

The test of a large panel of enteropathogenic and non-pathogenic *Yersinia* strains showed that SSI incubated at 37°C allowed the efficient growth of all 43 pathogenic *Y. enterocolitica* strains tested, while moderately to severely inhibiting the development of several non-pathogenic species such as *Y. bercovieri*, *Y. mollaretii* and *Y. intermedia*. CIN also allowed the efficient growth of all pathogenic *Y. enterocolitica* strains tested at 28°C, the recommended temperature for this medium, but had no inhibitory effect on non-pathogenic species. As the aim of the study was to evaluate SSI for the isolation of *Yersinia* at the temperature commonly used for other enterobacteria (37°C), the results obtained on SSI at 28°C will not be discussed. Under these chosen conditions, SSI would thus have the advantage over CIN of inhibiting the growth of some non-pathogenic *Yersinia* strains.

CIN has been shown to have an inhibitory effect on the growth of some *Y. pseudotuberculosis* strains [26], [30], [34]. We did observe in this study that 16 out of the 24 *Y. pseudotuberculosis* strains tested had a moderate to severe growth defect (from 1 to 4 logs) upon growth on CIN at the recommended temperature of 28°C. *Y. pseudotuberculosis* is not a frequent human pathogen in most countries worldwide [35], [36]. However, the low reported incidence of human pseudotuberculosis might not only be the consequence of a low rate of infection, but also of a poor efficiency of recovery of the pathogen from the feces, due to the lack of an appropriate isolation medium. We wondered whether SSI could be more suitable for the isolation of this species. This was not the case, as SSI exerted an even stronger inhibitory effect on all 24 *Y. pseudotuberculosis* strains tested upon incubation at 37°C. SSI is thus even less suitable than CIN for the recovery of *Y. pseudotuberculosis* from biological samples.

It has been previously reported [26], [30], [34], and we also found in this study, that enteropathogenic and non-pathogenic *Yersinia* colonies have a similar aspect on CIN, although non-pathogenic colonies are usually slightly larger. We observed the same phenomenon on SSI plates at 37°C: pathogenic *Y. enterocolitica* colonies had a shape and color similar to those of non-pathogenic strains, but a tendency to be smaller, although a certain degree of heterogeneity in size within a pure bacterial population was observed. These preliminary results thus suggested that despite a more potent inhibitory effect on *Y. pseudotuberculosis*, SSI incubated at 37°C had the same capacity as CIN at 28°C to grow pathogenic *Y. enterocolitica* and to distinguish them from non-pathogenic strains, based on the size of their colonies. Since *Y. enterocolitica* is the largely predominant enteropathogenic *Yersinia* species in most countries over the world, and since some clinical pathology laboratories are reluctant to use CIN, it was still interesting to determine whether a unique stool culture procedure using SSI incubated at 37°C could replace a *Yersinia*-specific procedure for the isolation of pathogenic *Y. enterocolitica* from stools.

The next step was then to compare the capacity of CIN and SSI to recover pathogenic *Y. enterocolitica* when mixed with other bacterial species. CIN has been shown to inhibit the growth of most bacteria but to be permissive for some species belonging to the genera *Acinetobacter, Aeromonas, Citrobacter, Enterobacter, Morganella, Pseudomonas* and *Serratia* [30], [37]. Furthermore, colonies of *C. freundii*, *Enterobacter agglomerans* and *Serratia liquefaciens* are not reliably differentiated from those of *Y. enterocolitica* [37]. Nonetheless, the inhibition of most other bacterial species and the morphology of *Yersinia* colonies on CIN significantly improved their recovery from a poly-contaminated sample. Since SSI has been designed to allow the isolation of all pathogenic enterobacteria (except *Campylobacter*), it is by essence less selective than CIN. An easy differentiation of *Y. enterocolitica* colonies from other enterobacteria on SSI is thus required. It has been reported previously that *Yersinia* colonies have a morphology on SSI at 37°C that allows their differentiation from other common enterobacteria such as *C. freundii, Edwardsiella tarda, Enterobacter spp., E. coli, Klebsiella spp., M. morganii, Plesiomonas spp., Proteus mirabilis, Proteus vulgaris, Providencia spp., Salmonella spp., Shigella spp. and Vibrio spp.* [32]. In agreement with this study, we found here that colonies of *P. vulgaris*, *S. dysenteriae*, *C. freundii* or *S.* Typhimurium could easily be distinguished from those of *Y. enterocolitica*. Because of their pink color, *E. coli, C. sakazakii* and *K. oxytoca* colonies were more similar to those of *Y. enterocolitica*, but co-culture experiments showed that the smaller size and lighter pink color of the latter allowed its relatively easy differentiation from *E. coli* and *K. oxytoca*. This was less obvious for *C. sakazakii* colonies: when they were next to *Y. enterocolitica* colonies, their slightly bigger size and darker pink color allowed their distinction. However, because the differences were minor, it might not be easy to differentiate the two types of colonies in an undefined biological sample. Despite this possible misidentification, we thus found that, as reported, SSI could allow the distinction of *Y. enterocolitica* from most other enterobacteria.

The final and most important step was to evaluate the efficacy of SSI to recover *Y. enterocolitica* colonies in the presence of numerous microorganisms composing human fecal flora. We found that high numbers of probably non-pathogenic bacteria present in a normal human stool were able to grow on SSI, but not on CIN, suggesting that the isolation of *Y. enterocolitica* colonies among an abundant fecal flora might be difficult on SSI. We tested this hypothesis by artificially contaminating human stools with various quantities of *Y. enterocolitica*. A load of 3×10^6^ *Yersinia*/g of feces was required to detect positive colonies, and their morphology was not markedly different from that of other bacteria, except their smaller size. In contrast, CIN allowed the growth of an almost pure *Y. enterocolitica* population, and could still detect colonies at the highest dilution used (3 colonies in the inoculum, corresponding to 3×10^3^ cfu/g of feces). This demonstrates that CIN is much more efficient than SSI at isolating *Y. enterocolitica* from a poly-contaminated biological sample, exhibiting a 3 log higher sensitivity. Information about the *Y. enterocolitica* load in stools of infected patients is scarce, but using RT-PCR it has been recently reported that this load ranges from 10^5^ to 10^8^ cfu/g of feces [38]. Furthermore, after antibiotic treatment, almost one third of the patients still continued to carry between 10^3^ and 10^6^ *Y. enterocolitica* cfu/g of feces and developed secondary complications [38]. Given that PCR-based methods are more sensitive than culture methods and that PCR also detect dead bacteria, it can be predicted that a significant number of patients would not have been culture-positive on SSI plates. The detection limit of *Yersinia* on SSI was previously reported to be between 5×10^1^ and 5×10^2^ cfu/g of feces [32]. The discrepancies with the results obtained in this study may have at least two reasons. The first one is that the stool suspension used in the previous work was 10 times more concentrated (1 g/ml) than in our study (1 g/10 ml). Actually, we purposely used here the procedure of common practice in clinical pathology laboratories (i.e. one pea (≈1 g) of stool suspended in 10 ml of saline or PBS). A second possible reason for these discrepancies could be that the human stools that were artificially contaminated differed in their composition and abundance of Gram-negative bacteria. If so, this would mean that the efficacy of SSI to recover pathogenic *Y. enterocolitica* varies depending on the stool samples analyzed. Moreover, the previously reported detection limit of ≤5×10^2^ cfu/g [32] may seem surprisingly low since both the more selective CIN medium and the more sensitive RT-PCR method, had a detection limit ≥10^3^ cfu/g of feces (this study and [38]).

In conclusion, the aim of this study was to determine whether it was possible to identify all enteropathogenic bacteria including *Yersinia* with a single procedure based on SSI. The ultimate goal was to eliminate the cost and workload associated with the necessity of a specific *modus operandi* for *Yersinia* isolation, and therefore to increase the number of medical laboratories that would isolate enteropathogenic *Yersinia* through the use of a common procedure for enteropathogenic Gram-negative bacteria. Our results indicate that SSI is not usable for the isolation of *Y. pseudotuberculosis*. Furthermore, because of its lower selectivity against common enterobacteria, it is much less efficient than CIN at recovering pathogenic *Y. enterocolitica* colonies from stools. Therefore, although negative, this information is important for clinical pathology laboratories, as it shows that many *Yersinia* infections might not be detected if the SSI medium is used for their isolation. It also indicates that, although not very convenient from a practical point of view, the usual procedure (CIN medium incubated at 28°C) still remains the most effective method for the bacteriological diagnosis of human yersiniosis, and should thus continue to be used.

## Supporting Information

Figure S1 **Schematic description of the procedure used for the screening of a large number of** ***Yersinia*** **strains.**
(TIF)

Figure S2 **Streaking of a human stool artificially contaminated with** ***Y. enterocolitica*** **IP29492.** Approximately 3×10^4^ *Y. enterocolitica* cfu were streaked. The plates were incubated at 37°C for 24 h. All colonies picked on CIN were *Y. enterocolitica*. The arrows on SSI plates point to colonies that were confirmed to be *Y. enterocolitica*.
(TIF)

Table S1 ***Yersinia*** **strains tested in this study.** NAG: non-agglutinable; NA: not applicable; IP: strains from the collection of the *Yersinia* Research Unit/National Reference Laboratory; CIP: strains from the Collection of the Institut Pasteur. Superscript T means Type Strain.
(DOCX)

Table S2 **Growth of** ***Y. pseudotuberculosis*** **on SSI and CIN as compared to TSA (control medium).** ^a^: the results are expressed as the difference of the log dilution limit between TSA and CIN, or between TSA and SSI.
(DOCX)
