# Supplementary figures and images for: Evaluation of a Single Procedure Allowing the Isolation of Enteropathogenic Yersinia along with Other Bacterial Enteropathogens from Human Stools
Source: PLoS One. 2012 Jul 20;7(7):e41176. doi: 10.1371/journal.pone.0041176 (PMC3401097; doi:10.1371/journal.pone.0041176)

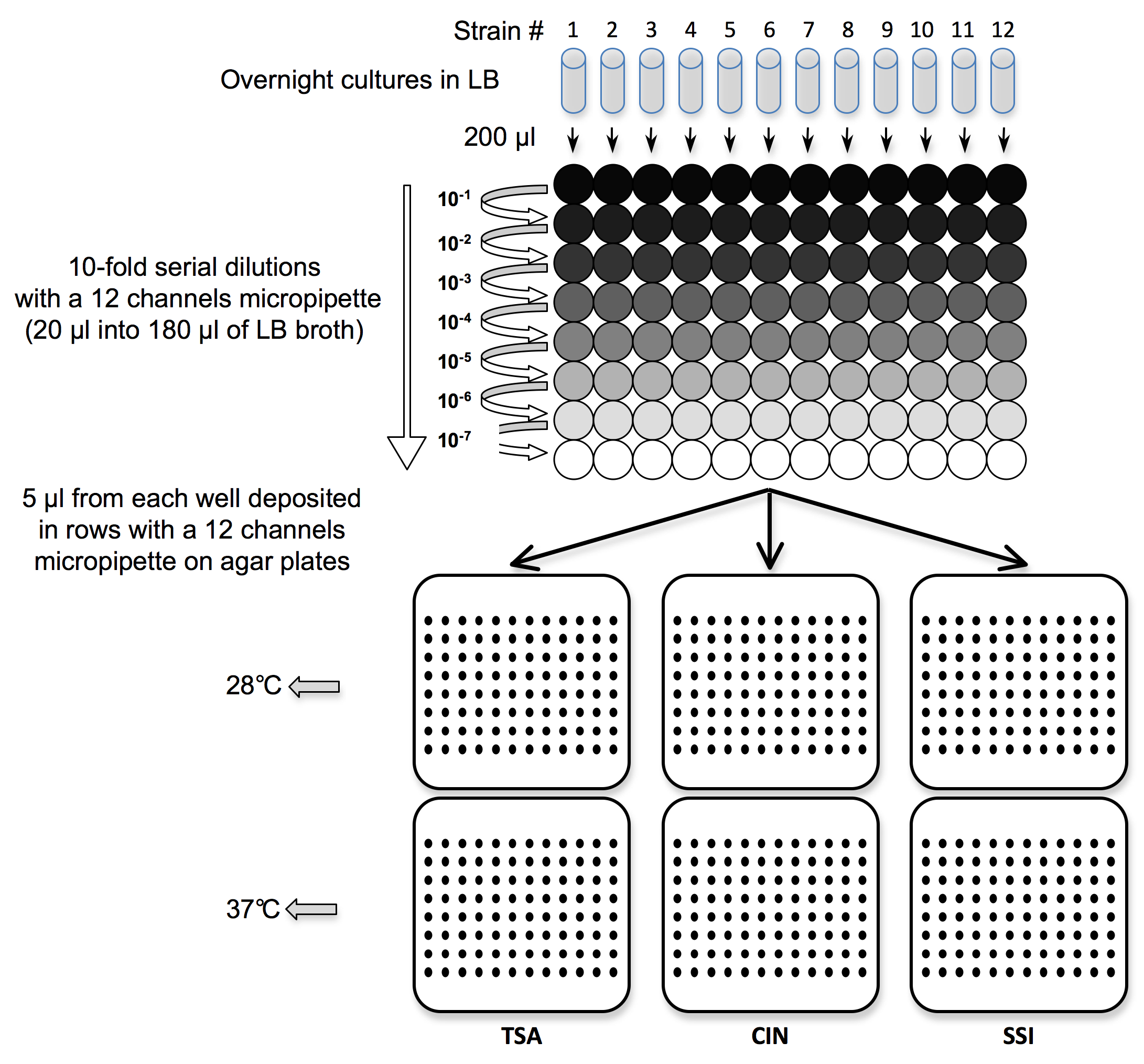

Supplement: Figure S1 — Schematic description of the procedure used for the screening of a large number of Yersinia strains. (TIF) [file pone.0041176.s001.tif]

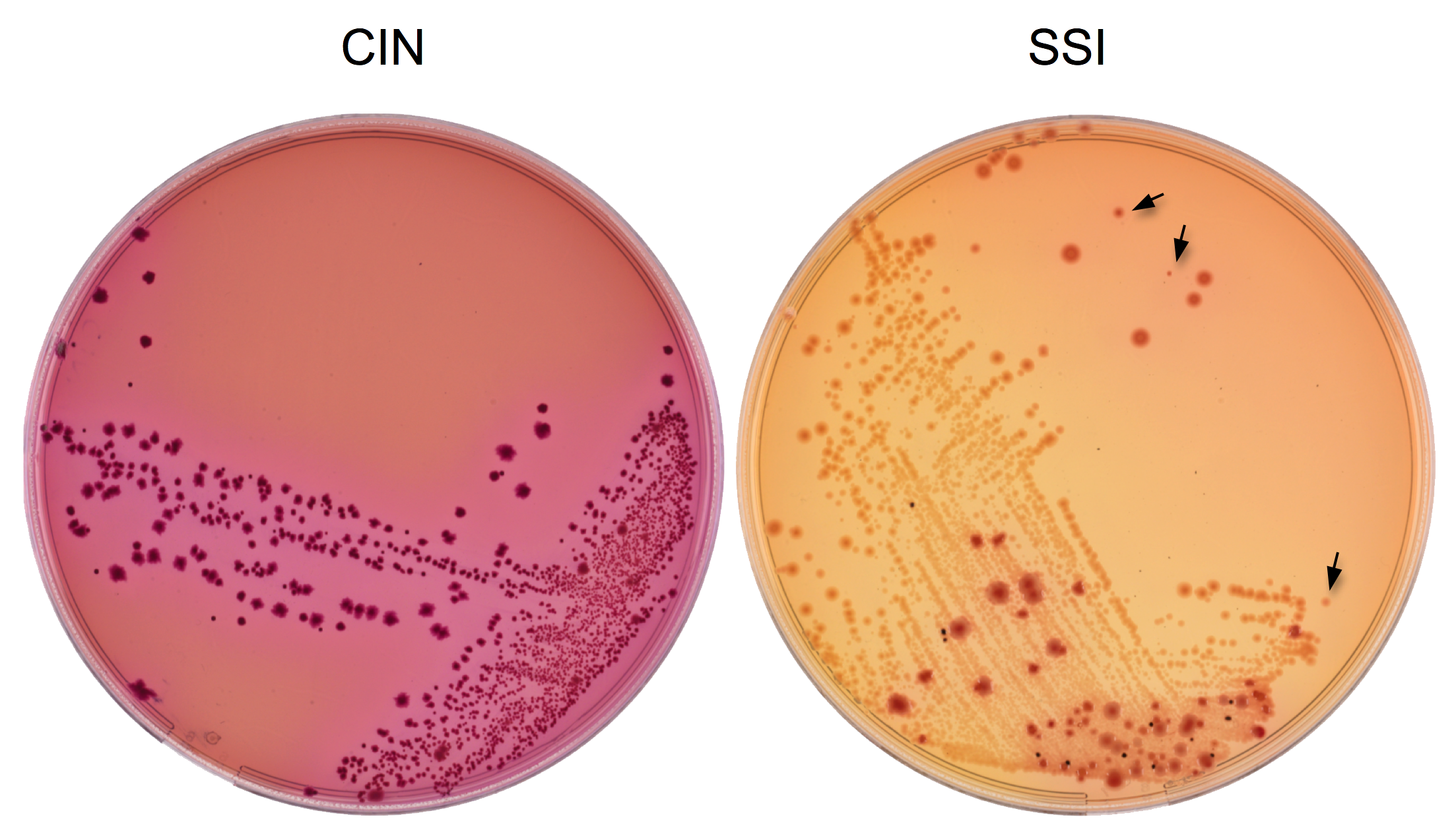

Supplement: Figure S2 — Streaking of a human stool artificially contaminated with Y. enterocolitica IP29492. Approximately 3×10^4^ Y. enterocolitica cfu were streaked. The plates were incubated at 37°C for 24 h. All colonies picked on CIN were Y. enterocolitica. The arrows on SSI plates point to colonies that were confirmed to be Y. enterocolitica. (TIF) [file pone.0041176.s002.tif]
