# Supplementary material for: Evaluation of a Single Procedure Allowing the Isolation of Enteropathogenic Yersinia along with Other Bacterial Enteropathogens from Human Stools
Source: PLoS One. 2012 Jul 20;7(7):e41176. doi: 10.1371/journal.pone.0041176 (PMC3401097; doi:10.1371/journal.pone.0041176)
Supplement: Table S1 — Yersinia strains tested in this study. NAG: non-agglutinable; NA: not applicable; IP: strains from the collection of the Yersinia Research Unit/National Reference Laboratory; CIP: strains from the Collection of the Institut Pasteur. Superscript T means Type Strain. (DOCX) [file pone.0041176.s003.docx]

Table S1. *Yersinia* strains tested in this study.

| **Species** | **Biotype** | **Serotype** | **Nb of strains** | **Strain number** |
| --- | --- | --- | --- | --- |
| *Y. enterocolitica* | 1A | NAG; O:12,25-12,26-25,35-35; O:18; O:41,42-41,43; O:6,30-6,31; O:4,32-4,33 | 6 | IP29470, IP29469, IP29468, IP29465, IP29463, IP29462 |
|  | 1B | O:8; O:5; O:8,19; O:16; O:41,42,43; O:20 | 10 | IP1105, IP14294, IP17451, IP19049, IP19052, IP19145, IP19831, IP19918, IP19958, Ye8081 |
|  | 2 | O:5,27 | 6 | IP29452, IP29434, IP29416, IP29388, IP28614, IP25597 |
|  | 2 | O:9 | 14 | IP24083, IP29235, IP29239, IP29305, IP29312, IP29313, IP29314, IP29345, IP29361, IP29382, IP29397, IP29403, IP29411, IP29497 |
|  | 3 | O:3 | 6 | IP29428, IP29394, IP29194, IP29159, IP28884, IP28877 |
|  | 4 | O:3 | 14 | IP29367, IP29368, IP29383, IP29385, IP29387, IP29393, IP29395, IP29396, IP29399, IP29401, IP29402, IP29410, IP29464, IP29492 |
|  | 5 | O:1,2,3 | 1 | IP26042 |
| *Y. pseudotuberculosis* | NA | I | 13 | IP33426, IP33427, IP33428, IP33429, IP33431, IP33433, IP33435, IP33436, IP33437, IP33438, IP33439, IP33440, IP33260 |
|  | NA | II | 6 | IP32554, IP32555, IP32576, IP32584, IP32596, IP32598 |
|  | NA | III | 5 | IP32544, IP32666, IP32887, IP33297, IP33277 |
| *Y. intermedia* | 2 | NAG, O:4,32-4,33-16-16,29 | 3 | IP29391, IP29407, IP29420 |
| *Y. frederiksenii* | NA | NAG | 2 | IP29330, IP29447 |
| *Y. kristensenii* | NA | NAG, O:16-16,29 | 2 | IP29386, IP29443 |
| *Y. bercovieri* | NA | NAG, O:4,33-16-16,29 | 2 | IP29390, IP29461 |
| *Y. mollaretii* | NA | NAG, O:7,8 | 2 | IP29024, IP29466 |
| *Y. aldovae* | NA | NAG | 1 | CIP103162^T^ |
| *Y. rohdei* | NA | NAG | 1 | CIP103163^T^ |

NAG: non-agglutinable; NA: not applicable; IP: strains from the collection of the *Yersinia* Research Unit/National Reference Laboratory; CIP: strains from the Collection of the Institut Pasteur. Superscript T means Type Strain.
