# Supplementary material for: Evaluation of a Single Procedure Allowing the Isolation of Enteropathogenic Yersinia along with Other Bacterial Enteropathogens from Human Stools
Source: PLoS One. 2012 Jul 20;7(7):e41176. doi: 10.1371/journal.pone.0041176 (PMC3401097; doi:10.1371/journal.pone.0041176)
Supplement: Table S2 — Growth of Y. pseudotuberculosis on SSI and CIN as compared to TSA (control medium). a: the results are expressed as the difference of the log dilution limit between TSA and CIN, or between TSA and SSI. (DOCX) [file pone.0041176.s004.docx]

Table S2. Growth of *Y. pseudotuberculosis* on SSI and CIN as compared to TSA (control medium).

| **Strain number** | **Serotype** | **Inhibitory effect of CIN or SSI^a^** | | | |
| --- | --- | --- | --- | --- | --- |
|  |  | **28°C** |  | **37°C** |  |
|  |  | **CIN** | **SSI** | **CIN** | **SSI** |
| IP33426 | I | 1 | 1 | 1 | 4 |
| IP33427 | I | 1 | 1 | 1 | 4 |
| IP33428 | I | 1 | 1 | 1 | 4 |
| IP33429 | I | 1 | 1 | 1 | 4 |
| IP33431 | I | 1 | 1 | 1 | 4 |
| IP33433 | I | 1 | 1 | 1 | 4 |
| IP33435 | I | 1 | 1 | 1 | 5 |
| IP33436 | I | 1 | 1 | 1 | 5 |
| IP33437 | I | 1 | 1 | 1 | 4 |
| IP33438 | I | 1 | 1 | 1 | 4 |
| IP33439 | I | 1 | 1 | 1 | 4 |
| IP33440 | I | 1 | 1 | 1 | 4 |
| IP33260 | I | 0 | 0 | 1 | 4 |
| IP32554 | II | 0 | 0 | 3 | 3 |
| IP32555 | II | 0 | 0 | 3 | 4 |
| IP32576 | II | 0 | 0 | 1 | 4 |
| IP32584 | II | 0 | 0 | 0 | 3 |
| IP32596 | II | 4 | 0 | 4 | 4 |
| IP32598 | II | 1 | 0 | 3 | 4 |
| IP32544 | III | 0 | 2 | 2 | 2 |
| IP32666 | III | 1 | 4 | 4 | 4 |
| IP32887 | III | 0 | 0 | 0 | 3 |
| IP33297 | III | 2 | 0 | 0 | 3 |
| IP33277 | III | 0 | 0 | 0 | 3 |

^a^: the results are expressed as the difference of the log dilution limit between TSA and CIN, or between TSA and SSI.
